# Standardization of blood smears prepared in transparent acetate: an alternative method for the microscopic diagnosis of malaria

**DOI:** 10.1186/1475-2875-13-238

**Published:** 2014-06-17

**Authors:** Marcia BC Mello, Francisco C Luz, Fabio A Leal-Santos, Eduardo R Alves, Thamires M Gasquez, Cor JF Fontes

**Affiliations:** 1 Laboratório de Malária, Hospital Universitário Júlio Müller, Rua Prof Luiz Phelippe Pereira Leite s/n Alvorada, Cuiabá, MT CEP:78048-250, Brazil; 2 Núcleo de Medicina Tropical, Universidade de Brasília, Brasília, DF, Brazil; 3 Curso de Biomedicina, Centro Universitário UNIVAG, Várzea Grande, MT, Brazil; 4 Faculdade de Ciências Biomédicas (FACIMED), Cacoal, RO, Brazil

**Keywords:** Malaria, Microscopy, Blood smears, Transparent acetate

## Abstract

**Background:**

Due to students’ initial inexperience, slides are frequently broken and blood smears are damaged in microscopy training, leading to the need for their constant replacement. To minimize this problem a method of preparing blood smears on transparent acetate sheets was developed with the goal of implementing appropriate and more readily available teaching resources for the microscopic diagnosis of malaria.

**Methods:**

Acetate sheets derived from polyester were used to standardize the preparation and staining of thin and thick blood smears on transparent acetate sheets. Thick and thin blood smears were also prepared using the conventional method on glass slides. The staining was conducted using Giemsa staining for the thick and thin smears.

**Results:**

Microscopic examination (1,000x) of the thin and thick blood smears prepared on transparent acetate produced high-quality images for both the parasites and the blood cells. The smears showed up on a clear background and with minimal dye precipitation. It was possible to clearly identify the main morphological characteristics of *Plasmodium*, neutrophils and platelets. After 12 months of storage, there was no change in image quality or evidence of fungal colonization.

**Conclusion:**

Preparation of thin and thick blood smears in transparent acetate for the microscopic diagnosis of malaria does not compromise the morphological and staining characteristics of the parasites or blood cells. It is reasonable to predict the applicability of transparent acetate in relevant situations such as the training of qualified professionals for the microscopic diagnosis of malaria and the preparation of positive specimens for competency assessment (quality control) of professionals and services involved in the diagnosis of malaria.

## Background

The main strategy for malaria control consists of early diagnosis and appropriate treatment. Delayed diagnosis or treatment contributes to the rapid spread of malaria and an increased risk of severe and fatal malaria cases [1]. In Brazil, the transmission of malaria predominantly occurs in the Amazon region in the north. The risk of succumbing to malaria in the non-endemic regions of this country, where malaria does not occur naturally, is 100 times greater for individuals who visit the Amazon than that for inhabitants of the Amazon region [2]. This is mainly due to diagnostic errors and delay in the treatment of malaria, because of inexperience of the professionals within these non-endemic regions with regard to the correct microscopic diagnosis of this infection. In fact, it is not rare for the disease to be diagnosed after death [3].

The microscopic analysis of a thickly stained blood smear is still the most suitable method for the diagnosis of malaria. It is a simple, quick, low-cost technique that enables the correct diagnosis of the malarial parasite species and the determination of parasite density. However, the lack of qualified professionals to correctly diagnose malaria and the lack of quality control in the laboratory diagnostic process have been identified as the main reasons for the lack of success in the current strategy to control this disease in Brazil [4].

To train professionals in obtaining a laboratory diagnosis of malaria, educational institutions, as well as services for the epidemiological surveillance of malaria throughout the country, provide practical classes and training for the microscopic diagnosis of *Plasmodium* spp. This practical teaching is essential for the training of biology, pharmacy and biomedical students and for assessing the expertise of technical professionals associated with diagnosis of malaria [5].

Microscopy training requires readily available and high-quality educational resources. Blood smears, including those positive for malaria, have been the most widely used educational resource in these classes. However, due to students’ initial inexperience, slides are frequently broken and blood smears are damaged, leading to the need for their constant replacement. In addition, replacing positive blood smears in practical classes involves their constant transport from the place where they were prepared to the location of the practical class. The suppliers of positive smears are found in areas where malaria is endemic, often far from the institutions that require them. Slides are transported by mail or carrier which is highly costly due to the weight of the glass; moreover, the slides are often broken during the collection, transportation and delivery processes.

To minimize this problem, based on a study by Luz [6], a method of preparing blood smears on transparent acetate sheets was developed with the goal of implementing appropriate and more readily available teaching resources for the microscopic diagnosis of malaria. Acetate is a generic term used to classify various plastics that appear similar to the naked eye but have different characteristics and different chemical compositions. These polymers are produced from crystalline polyvinyl chloride (PVC), polyphthalate polycarbonate (PPC), and polyethylene terephthalate (PET), with the latter being a thermoplastic polyester coated with silica that is used to absorb dyes in the printing processes.

## Methods

This is a descriptive study of all stages of the standardization and evaluation of a new health-related product that was designed to improve the teaching of malarial parasite morphology. This study was conducted in the Malaria Laboratory in the Infectious Diseases Clinic at Júlio Müller University Hospital from April 2012 to December 2013.

To standardize the preparation and staining of the blood smears on the transparent acetate sheets, blood from healthy laboratory staff members was used. Then, to analyse the quality of these blood smears for the diagnosis of malaria, blood samples were obtained from ten patients with malaria. The involvement of the individuals in this study was voluntary, and full consent was obtained. The study was approved by the Research Ethics Committee at Júlio Müller University Hospital as authorized by nº 171082 on 12 December, 2012.

PPC acetates obtained from different manufacturers and only one crystalline PVC brand name were tested with regard to their resistance in contact with methanol, which is used to fix blood smears. Small strips of each acetate sheet were immersed in test tubes filled with absolute methanol (Sigma-Aldrich, cat. M1775-1GA) and observed for 30 minutes. The acetate strip was then removed from the tube, and its texture and transparency were observed with the naked eye.

### Preparation of blood smears

**Figure 1:**
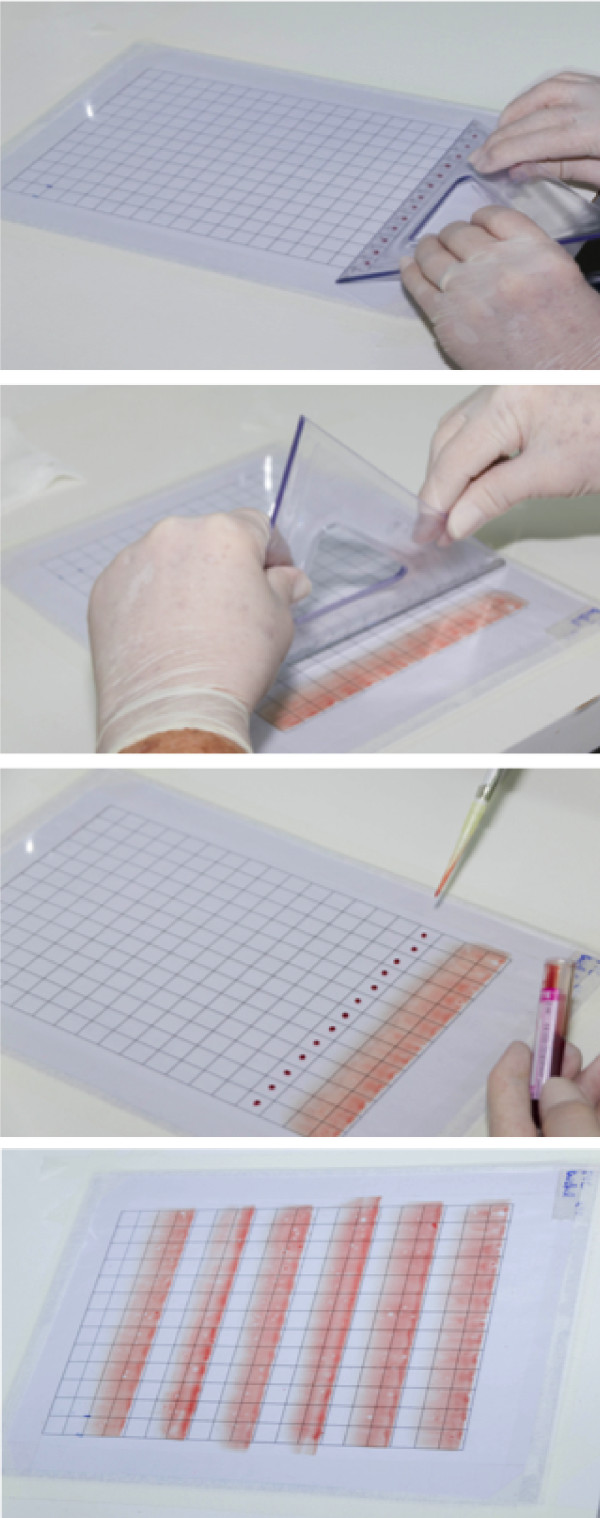
Steps for preparing a thin smear on a transparent acetate sheet.

**Figure 2:**
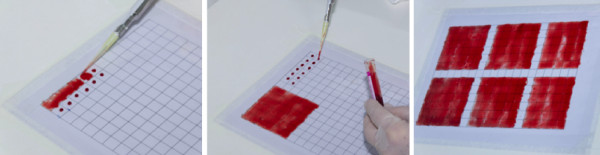
Steps for preparing a thick smear on a transparent acetate sheet.

The acetate sheet was previously treated with gauze soaked in 96% absolute alcohol to guarantee the homogenous distribution of blood in the smear. First, an A4 paper sheet was printed with a grid table containing 273 uniform 1.2 sq cm cells. The acetate sheet was then overlapped the paper sheet, in order to guide the space to be filled by blood smear (Figures 1 and 2). Immediately after the blood was collected, thin and thick blood smears were prepared on glass slides and transparent acetate sheets.Using an automatic pipette, 4 μL of blood was dropped onto the acetate, at 1.2-cm intervals. With a medium-sized acrylic square, the blood was spread out vertically over the sheet to form a thin smear with approximately 3.6 cm length and 15.6 cm wide. This process was repeated in the remaining space on the acetate sheet (Figure 1). For the thick smear, 10 μL of blood was dropped onto the acetate at 1.2-cm intervals. The blood was then spread out using a rod to cover a larger area of acetate. Better homogenization of the thick smear was obtained when the blood spreading was limited to rectangles of approximately 50–90 sq cm (Figure 2). Only one patient sample was used per acetate sheet in order to prevent cross-contamination.

The sheets with the blood smears were kept in a dry environment and protected from dust and insects for 12–24 hours, after which the thin blood smears were fixed with methanol. Thick and thin blood smears were also prepared using the conventional method on glass slides [7] previously treated with 96% absolute alcohol as well.

### Staining of blood smears

After drying, the thick blood smears were rapidly dehaemoglobinized with an aqueous solution of 0.4% methylene blue (Labsynth™, Labsynth Produtos Laboratório Ltda, São Paulo, Brazil), applied using a dispensing bottle, and then rinsed in buffered water. This rapid staining stage with methylene blue was added to increase the contrast of the parasites upon the debris of the haemolysed blood cells in the thick smear [5].

**Figure 3:**
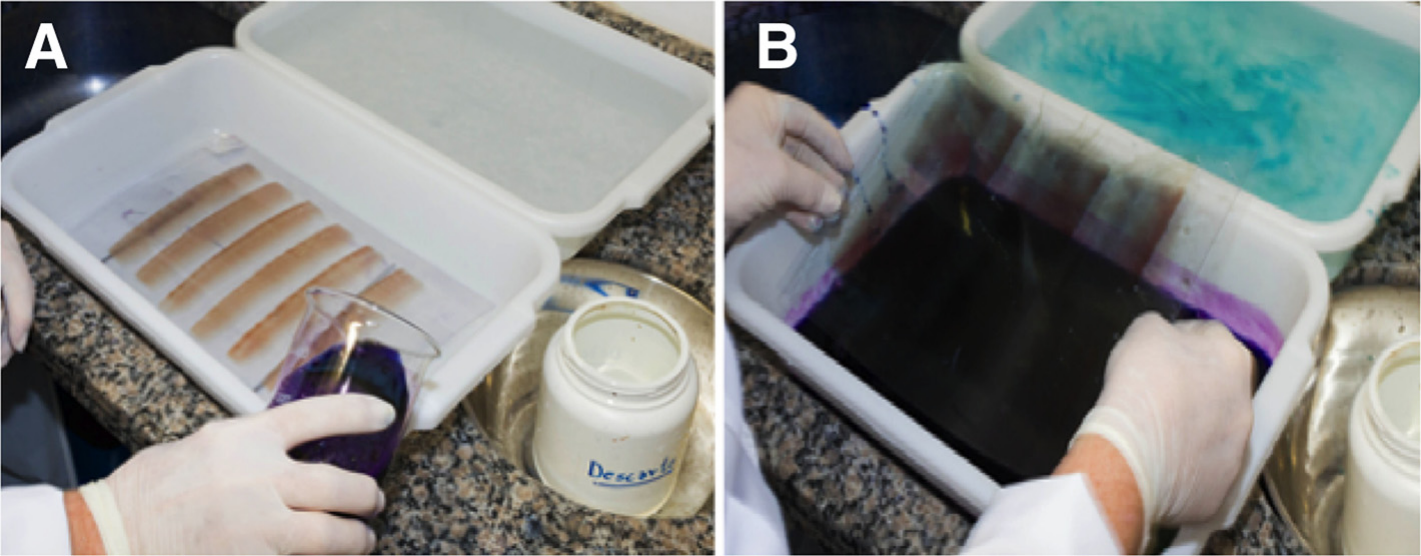
Tray staining of a thin (A) and thick smear (B) on a transparent acetate sheet.

**Figure 4:**
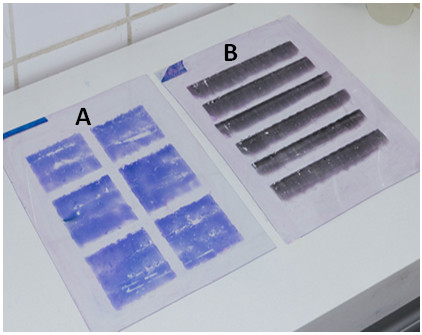
Thick (A) and thin (B) blood smears prepared on acetate sheets after staining.

**Figure 5:**
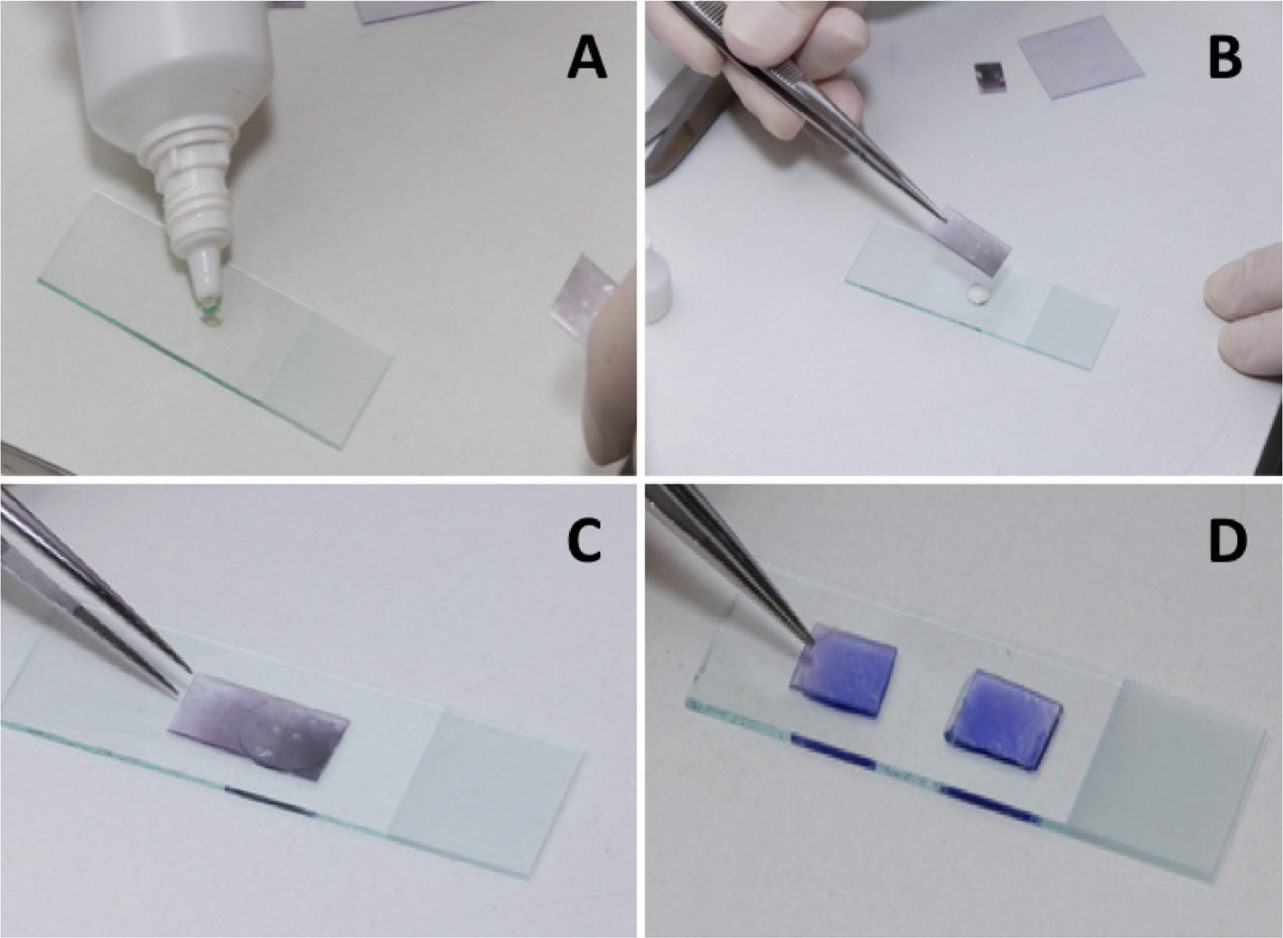
**Mounting of a smear fragment in acetate upon a glass slide: a drop of immersion oil is added (A) and the acetate fragment is placed on top (B).** The slide is mounted with a thin **(C)** and thick **(D)** smear fragment.

The acetate sheets were then placed on a plastic tray on two metal rods for support. This ensured that the entire length of the smear on the acetate was simultaneously stained. To stain the two acetate sheets, ie, the thick and thin smears, approximately 300 mL of 10% diluted Giemsa solution (Labsynth™, Labsynth Produtos Laboratório Ltda, São Paulo, Brazil) was used (Figure 3A and B). For acetate sheets and glass slides, the staining method was the same and was performed as recommended by WHO [7,8]. An example of the final results of the stained smears on acetate is shown in Figure 4.Once stained, the acetate sheets were cut into rectangular strips and mounted on glass slides. A drop of immersion oil was used to fix the acetate strips to the glass slide according to the sequence shown in Figure 5.

### Fixation of blood smears with finishing varnish and mounting on glass slides

**Figure 6:**
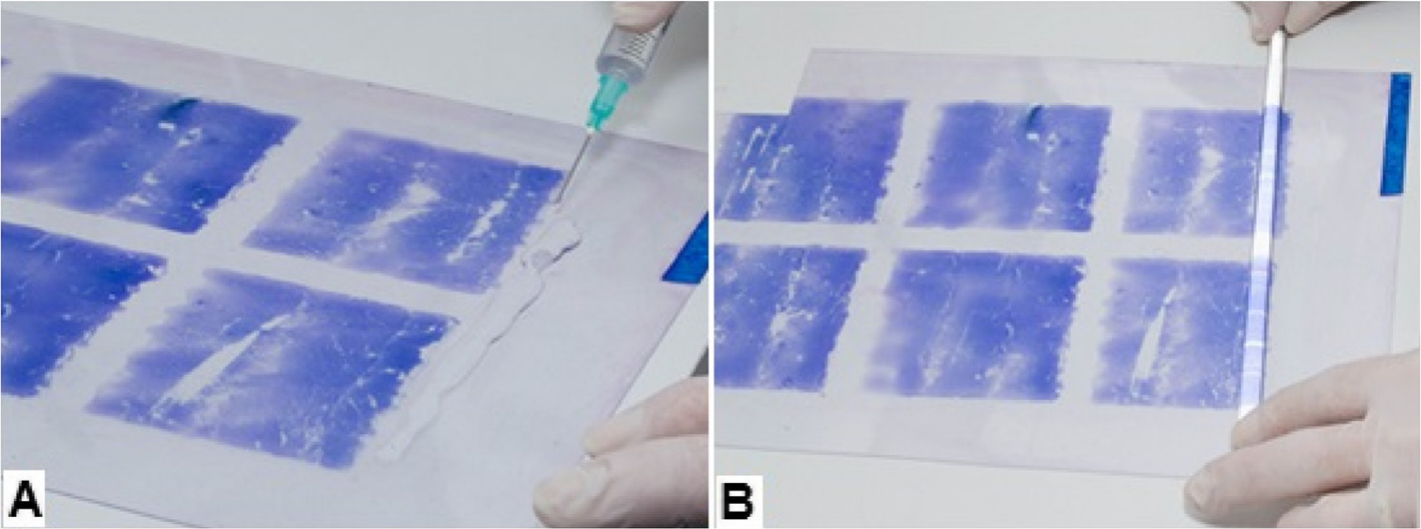
Applying of the finishing varnish over the blood smear prepared on acetate sheet using a syringe (A) and spreading it with a glass rod (B).

After staining, the blood smears prepared on acetate were manually coated with a finishing varnish (DuPont®, São Paulo – SP, Brazil). Using a 5 ml syringe attached to a 27 gauge needle, a line of the varnish was manually applied to the edge of the acetate sheet. By sliding a glass rod over the sheet, the varnish was spread across all the smear (Figure 6). The blood smears were fixed to increase durability and protect them from possible damage or colonization by fungi and bacteria. The drying time of this varnish was 5–10 minutes.

After fixation, the acetate sheets were cut into small rectangular strips (1.2 sq cm for thick blood smears and 2.4 sq cm for thin blood smears). The acetate strip was then placed onto a glass slide atop a drop of immersion oil for fixation. No coverslips were used.

### Evaluation of quality and durability of blood smears prepared on transparent acetate

Verifying the quality of blood smears prepared on acetate involves analysing the staining and morphology of parasites and blood cells under the microscope compared with blood smears mounted on glass slides. These images were photodocumented using a Zeiss-Scope-A1 microscope (Carl-Zeiss, Oberkochen, Germany). Moreover, all of the blood smears were analysed by three microscopists to estimate the parasitic density in each patient. The different blood stages of the parasites were identified from 200 microscopic fields (1,000×) and the parasitaemia was estimated by multiplying the amount recorded by a factor of 2.5 since this microscopic area is approximately 0.4 cu mm of blood [9]. The F-test and the Bartlett test were used to compare the variances between the parasite densities by the three microscopists with the significance level set at 95%.

The durability of the blood smears mounted on transparent acetate was evaluated based on the persistence of the quality characteristics evaluated above and the absence of fungal colonisation after the specimens were stored for a period of 12 months.

### Possibility of reusing the smear mounted on acetate

Some glass slides were deliberately broken to simulate everyday accidents during malaria microscopy training sessions. The acetate strip containing the smear was recovered and reassembled onto a new glass slide. The preparation and staining quality of the recovered smear were then evaluated as described above.

## Results

Testing was conducted on 11 types of acetate sheets derived from polyester, which in practice are used as model artefacts or transparency in photocopiers and printers. Only five commercial acetates made of PPC or crystalline PVC resisted treatment with methanol and were used in other standardization procedures. Information on the 11 acetate sheets tested is shown in Table 1.

**Table 1:** Information on the acetate sheets tested for their resistance in contact with methanol

**Chemical composition**	**Trade mark**	**Usefulness**	**Resistance to methanol**	**Manufacturer**
PVC	Adina	Architectural model makin	Yes	Adina Indústria e Comercio de Fechos, Rio de Janeiro, Brazil
PPC	Mares	Laser printing	Yes	Mares Comercial Ltda, São Paulo, Brazil
PPC	USA-Folien	Laser printing	Yes	USA Folien Brazil Ltda, São Paulo, Brazil
PPC	Garra	Laser printing	Yes	Industria Brasileira de Audiovisual Ltda, Blumenau, Brazil
PPC	3 M	Laser printing	Yes	3 M do Brasil, São Paulo, Brazil
PET	Premium Mares	Inkjet printing	No	Mares Comercial Ltda, São Paulo, Brazil
PET	USA-Folien	Inkjet printing	No	USA Folien Brazil Ltda, São Paulo, Brazil
PET	Garra	Inkjet printing	No	Industria Brasileira de Audiovisual Ltda, Blumenau, Brazil
PET	3 M	Inkjet printing	No	3 M do Brasil, São Paulo, Brazil
PET	Bellimagem	Inkjet printing	No	
PET	Unknown	Inkjet printing	No	Dunort Papelaria, Cuiabá, Brazil
PET	Unknown	Inkjet printing	No	Coxipó Papelaria, Cuiabá, Brazil

Three types of varnish were tested to fix the smears (Acrilex™, Acrilex Tintas Especiais SA, São Paulo, Brazil; Corfix™, Indústria de Tintas Corfix Ltda, São Paulo, Brazil; DuPont-Duxone DX6000™, Dupont do Brasil, São Paulo, Brazil). Only the varnish DuPont-Duxone DX6000™ gave a satisfactory result due to its rapid drying and lack of interfering in the blood smear staining or structure. The other two varnishs reacted with the immersion oil and damaged the blood smear.

**Figure 7:**
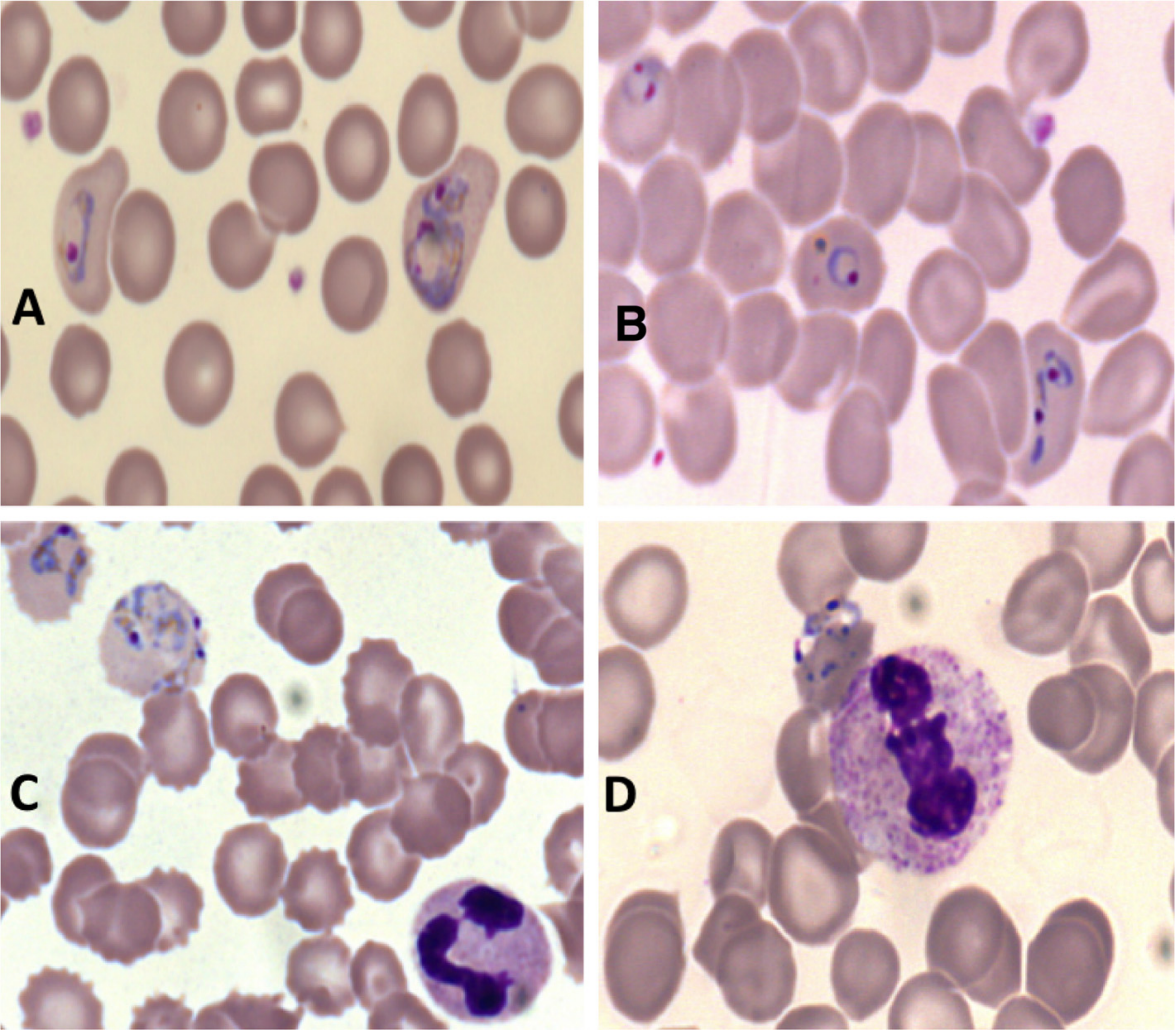
Microscopic images of the parasites and blood cells in the thin blood smears prepared on glass slides (A, C) and on a transparent acetate sheet (B, D).

**Figure 8:**
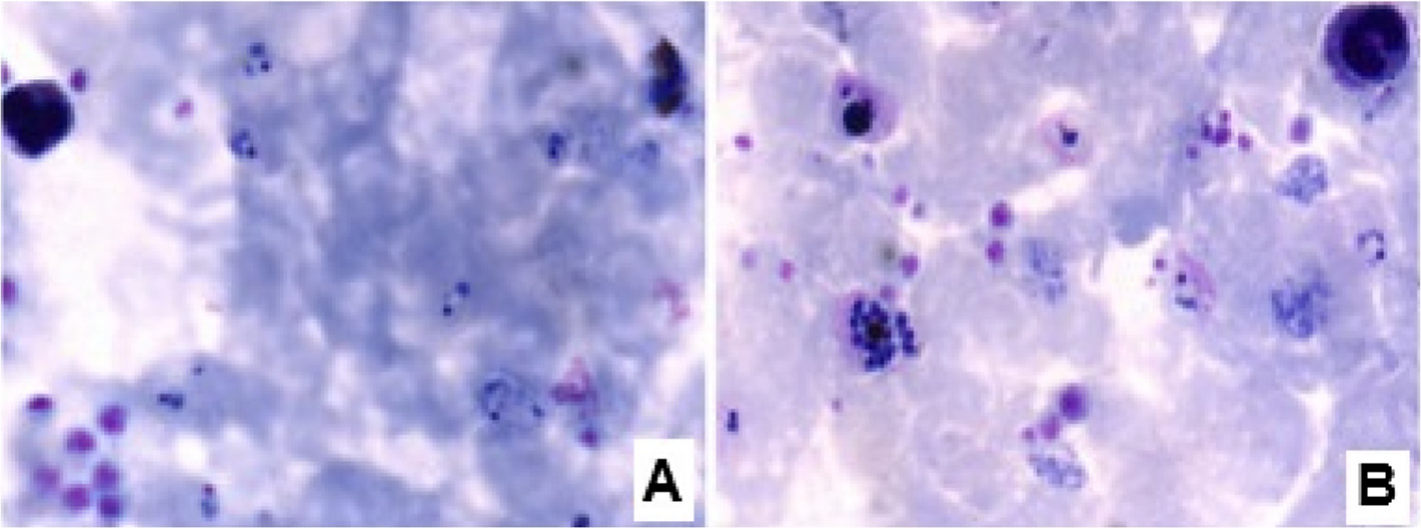
Microscopic images of the parasites and blood cells in the thick blood smears prepared on glass slides (A) and on transparent acetate sheet (B).

On a single acetate sheet, 78 (2.4 sq cm) strips with thin blood smears were prepared and 204 (1.2 sq cm) strips with thick blood smears (Figures 1 and 2). Microscopic examination (1,000×) of the thin and thick blood smears prepared on glass slides and transparent acetate sheets produced high-quality images for both the parasites and the blood cells. The smears mounted on acetate showed up on a clear background and with minimal dye precipitation. It was possible to clearly identify the main morphological characteristics of *Plasmodium*, which include chromatin (stained red), the Schüffner’s granules of *Plasmodium vivax* (pink), and faint cytoplasmic granules of neutrophils. Other characteristics, such as platelets (bright pink), leukocyte nuclei (dark), remaining blood cells (light blue), and chromatin of the parasites (red) confirmed the quality of the material for diagnosing malaria (Figures 7 and 8). After 12 months of storage, there was no change in image quality or evidence of fungal colonisation. In addition, when the acetate strip was recovered from the deliberately broken glass slides and examined again under the microscope, the image remained unchanged.

The parasites were counted by three microscopists and the results were concordant with a high equality of variance in parasite enumeration regardless of whether the samples were mounted on glass slides or transparent acetate sheets (Table 2).

**Table 2:** **Comparison of parasitic density** (***Plasmodium vivax*** **infection**) **from thick blood smears prepared on different media according to the microscopists**

**Smear support material**		**Microscopist (parasites/cu mm of blood)**		
		**A**	**B**	**C**
Glass slide	Mean	10,260	9,822	8,540
	Variance	63,616,000	69,581,045	53,139,333
	Standard deviation	7,976	8,342	7,290
Transparent acetate	Mean	10,220	8,436	8,030
	Variance	72,177,889	61,607,552	41,633,444
	Standard deviation	8,496	7,849	6,452
	p value*	0.712	0.870	0.991
	p value**	0.861	0.724	0.855

*F-test (analysis of variance); **Bartlett test for equality of variance.

## Discussion

In this study, the methodology for preparing thin and thick blood smears on transparent acetate for the microscopic diagnosis of malaria was described. This material is made of polyvinyl chloride or polyethylene terephthalate, which, in practice, is widespread in printers and photocopiers to print transparent films or even in the assembly of models by professional architects.

Preparing blood smears on glass slides for the diagnosis of malaria was initially presented during the Brazilian Society Congress of Tropical Medicine in João Pessoa (PB) in 1975. In this first experiment, the author proposed the use of the transparent material used in overhead projectors without describing the technical processes, standardization or evaluation of this new method of preparing smears [6]. Subsequently, an attempt to reproduce this method was described in a monograph that reinforced the findings described above; this suggested that transparent acetate was a sufficient support for blood smears used in the microscopic examination of malaria [10].

The alternative method for preparing blood smears on acetate sheets proved to be easy to conduct and did not require additional expenditure since the acetate sheets are inexpensive and easily found in local stationery shops. In addition, changing the smear support from glass to acetate did not jeopardize the microscopic image quality of the different stages of the malarial parasite or the blood cells. Even with a bluish tint to the background of the blood smears mounted on acetate, the morphologic and staining characteristics of the parasites and blood cells were not altered; this enabled their easy identification. The parasitaemia recorded by the three microscopists, who analysed both blood smears (glass and acetate), were highly consistent and showed that this alternative method does not result in the loss of parasites during mounting and staining. This was already observed by other authors in conventional smears [11].

Direct visualization of parasites under the microscope is still the most commonly used method worldwide for the diagnosis of malaria and is the main strategy for controlling the transmission and prevention of serious and complicated cases of malaria [1,4,12]. Therefore, successful control of malaria in endemic countries ultimately depends of the expertise of the professionals involved in the microscopic examination of malaria [12].

The competence and ability of professionals regarding the correct microscopic diagnosis of malaria are formed in practical microscopy lessons on blood smears. Teachers face several challenges when preparing and delivering these practical classes. First, slides containing the blood smears are not commercially available; the availability of positive slides depends on the willingness of staff living in endemic areas with access to patients to prepare the slides. Second, the slides require transportation, usually by mail, which often results in partial or complete breakage. Third, since students are generally inexperienced with microscopes, they frequently damage the blood smears or break the glass slides. In consequence, a common complaint among professors is the frequency with which the stock of slides with positive malaria smears needs to be replaced.

The availability of blood smears on acetate sheets prepared by the staff living in malaria endemic areas can minimize these difficulties. A large blood smear can be prepared from a small volume of blood positive with *Plasmodium* on a single acetate sheet, which produces a large number of stained samples to be mounted on various slides. If a glass slide containing the smear mounted on acetate breaks, the specimen is quickly recovered and reused, reducing the need to restock the slides for teaching purposes.

An advantage of preparing blood smears on acetate is due to the simplicity and convenience of their transportation; they can be placed in normal envelopes without any risk of damage during shipment. From a single A4 acetate sheet weighing <10 g, a professor will be able to prepare 204 thick blood smears or 78 thin blood smears. The same number of samples prepared on glass would result in a weight of approximately 1.5 kg.

Another potential advantage of blood smears being prepared on transparent acetate is that it provides diagnostic assistance to healthcare professionals in non-endemic areas who have little experience in the microscopic analysis of malaria. When faced with a patient with suspected malaria and in the lack of a malaria rapid diagnostic test, a blood smear on acetate can be prepared on-site and sent by courier to a reference centre. It is assumed that in <36 hours, a suspected malarial diagnosis can be confirmed, thus contributing to the prevention of serious and complicated cases.

Based on the advantages described above, the potential of blood smears prepared on acetate cannot be disregarded for assessing the competency and diagnostic quality control of the professionals and laboratories attending patients with malaria. External quality assessment tools are recognised by the World Health Organization and are essential for successfully controlling malaria in endemic areas [13]. The light weight, high quality and durability of this new blood smear will allow the preparation of a large number of positive smears for periodic submission to evaluation in external quality assessment programmes.

The reduced cost of preparing blood smears on transparent acetate was also evident in this study. The staining of 282 thick and thin blood smears prepared on acetate used only 300 mL of the diluted Giemsa solution. To prepare the same number of blood smears on glass slides, approximately 850 mL of the same solution would be required [8]. Therefore, it can be inferred that there is approximately a three-fold reduction in dye consumption, which contributes greatly to the financial cost of staining.

The time saved during the preparation of blood smears on acetate sheets must be highlighted. Even when provided with sufficient support for staining blood smears on glass slides, the time dedicated to fix and stain these slides would be no less than 3 h given that it takes 30 min to mount and stain about six sets of 50 glass slides. After the method has been standardized, the time required to stain the 282 smears in this study was less than one hour.

## Conclusions

Transparent acetate is an appropriate material for the preparation of thin and thick blood smears for the microscopic diagnosis of malaria. This material enables the simple and low-cost preparation of samples and does not compromise the morphologic and staining characteristics of the parasites or blood cells. Considering all of these advantages, it is reasonable to predict the applicability of transparent acetate in relevant situations such as the training of qualified professionals for the microscopic diagnosis of malaria, the preparation of positive specimens for competency assessment (quality control) of professionals and services involved in the diagnosis of malaria, and provision of diagnostic aid to healthcare professionals in non-endemic areas who have little experience in the microscopic diagnosis of malaria.

## Abbreviations

PVC: Crystalline polyvinyl chloride; PPC: Polyphthalate polycarbonate; PET: Polyethylene terephthalate.

## Competing interests

The authors declare that they have no competing interests.

## Authors’ contributions

FCL conceived the method. CJFF aided the study design and drafted the manuscript. MCMF supervised the laboratory work and revised the manuscript. ERA, FAL and TMG participated in the microscopic analysis of the blood smears. All authors read and approved the final manuscript.

## References

[B1] WHO Guidelines for the treatment of malaria 2010 2 Geneva: World Health Organization 25473692

[B2] Marcondes CB Marchi MJ Are extra-Amazonian physicians prepared to diagnose and treat malaria? Rev Soc Bras Med Trop 2010 43 477 10.1590/S0037-86822010000400032 20802958

[B3] Krettli AU Aguiar ACC Souza NB Chaves KM Fontes CJF Tauil PL Malaria fora da Amazônia Ciencia Hoje 2013 51 32 34

[B4] Oliveira-Ferreira J Lacerda MVG Brazil P Ladislau JLB Tauil PL Daniel-Ribeiro CT Malaria in Brazil: an overview Malar J 2010 9 115 10.1186/1475-2875-9-115 20433744 PMC2891813

[B5] Brasil. Ministério da Saúde Manual de diagnóstico laboratorial da malária 2009 Ministério da Saúde: Brasília-DF

[B6] Luz FCO Sociedade Brasileira de Análises Clínicas Obtenção de coleções de lâminas de plástico com P. falciparum, P. vivax e P. malariae Proceedings of the IV Congresso da Sociedade Brasileira de Análises Clínicas: June 1975; João Pessoa 1975 80 81

[B7] WHO Basic malaria microscopy Learner’s guide 2010 2 Geneva: World Health Organization

[B8] WHO Manual of basic techniques for a health laboratory 2003 2 Geneva: World Health Organization

[B9] Trape JF Rapid evaluation of malaria parasite density and standardization of thick smear examination for epidemiological investigations Trans R Soc Trop Med Hyg 1985 79 181 184 10.1016/0035-9203(85)90329-3 3890280

[B10] Leal-Santos FA Microscopia em distendido sanguíneo espesso preparado em acetato transparente: alternativa viável pata fins didáticos do ensino da parasitologia da malária. Graduate thesis 2010 Cuiabá: Instituto de Biociências da Universidade Federal de Mato Grosso

[B11] O'meara WP Mckenzie FE Magill AJ Forney JR Permpanich B Lucas C Gasser Jr RA Wongsrichanalai C Sources of variability in determining malaria parasite density by microscopy Am J Trop Med Hyg 2005 73 593 598 16172488 PMC2500224

[B12] WHO World Malaria Report 2012 2012 Geneva: World Health Organization

[B13] WHO Policy and procedures of the WHO/NICD microbiology external quality assessment programme in Africa 2007 Geneva: World Health Organization WHO/CDS/EPR/LYO/2007.3

